# Supplementary material for: Survival of the weakest in non-transitive asymmetric interactions among strains of E. coli
Source: Nat Commun. 2020 Nov 27;11:6055. doi: 10.1038/s41467-020-19963-8 (PMC7699631; doi:10.1038/s41467-020-19963-8)
Supplement: Supplementary file 1 — Supplementary Information [file 41467_2020_19963_MOESM1_ESM.pdf]

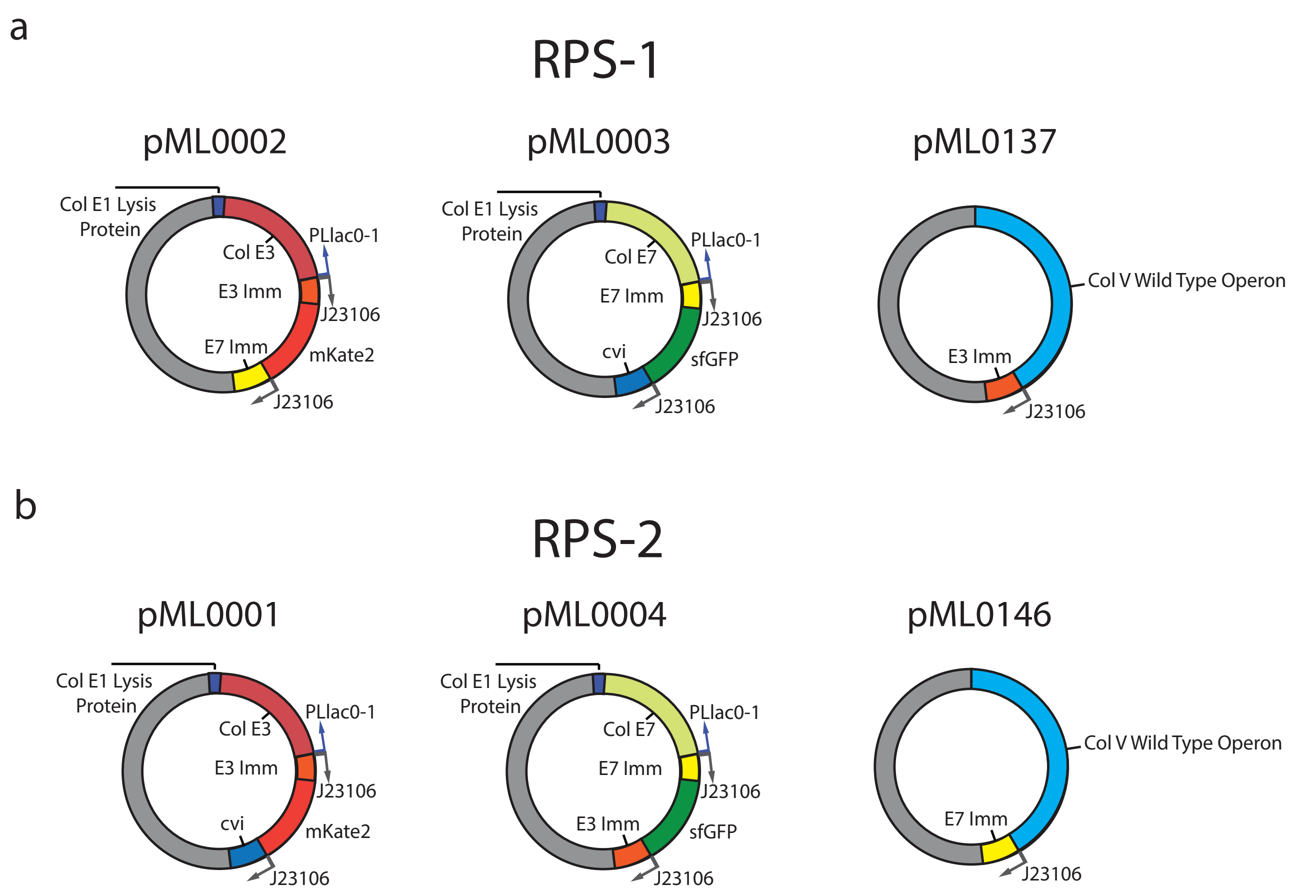

Supplementary Figure 1 | The plasmids used in this study(**a,**) Plasmids maps for strains R,G,B respectively. (**b,**) Plasmids maps for strains R2,G2,B2 respectively.

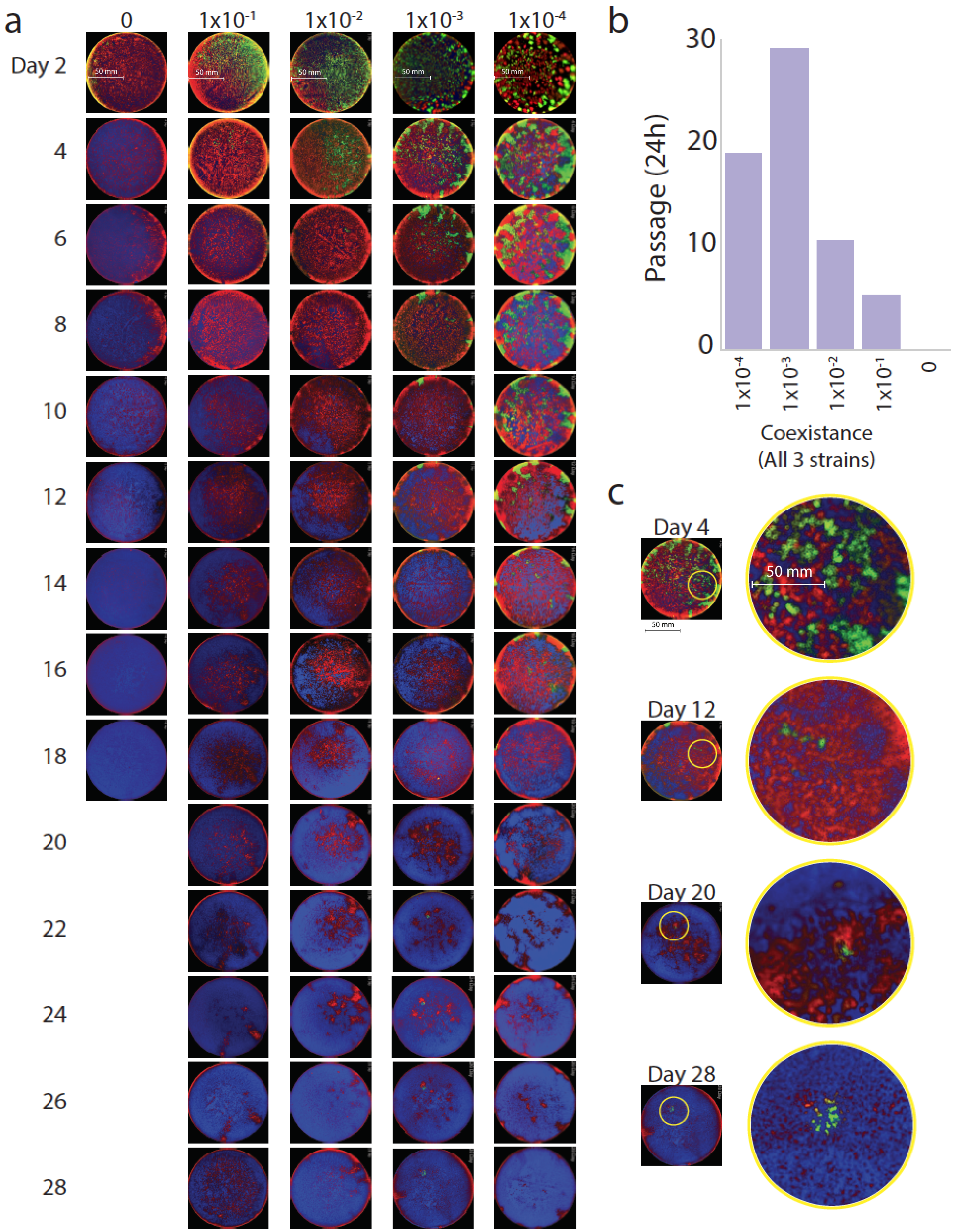

Supplementary Figure 2 | Replica plates of all serial dilution densities tested (**a,**) Images of combined three strain plate passages across a range initial starting densities. These experiments were executed one time for each starting density. (**b,**) Duration of three strain coexistence for each starting density. (**c,**) Closeup images of the highlighted regions on day 4, day 12, day 20, and day 28 of the $1x10^{-4}$ demonstrating the maintenance of coexistence.

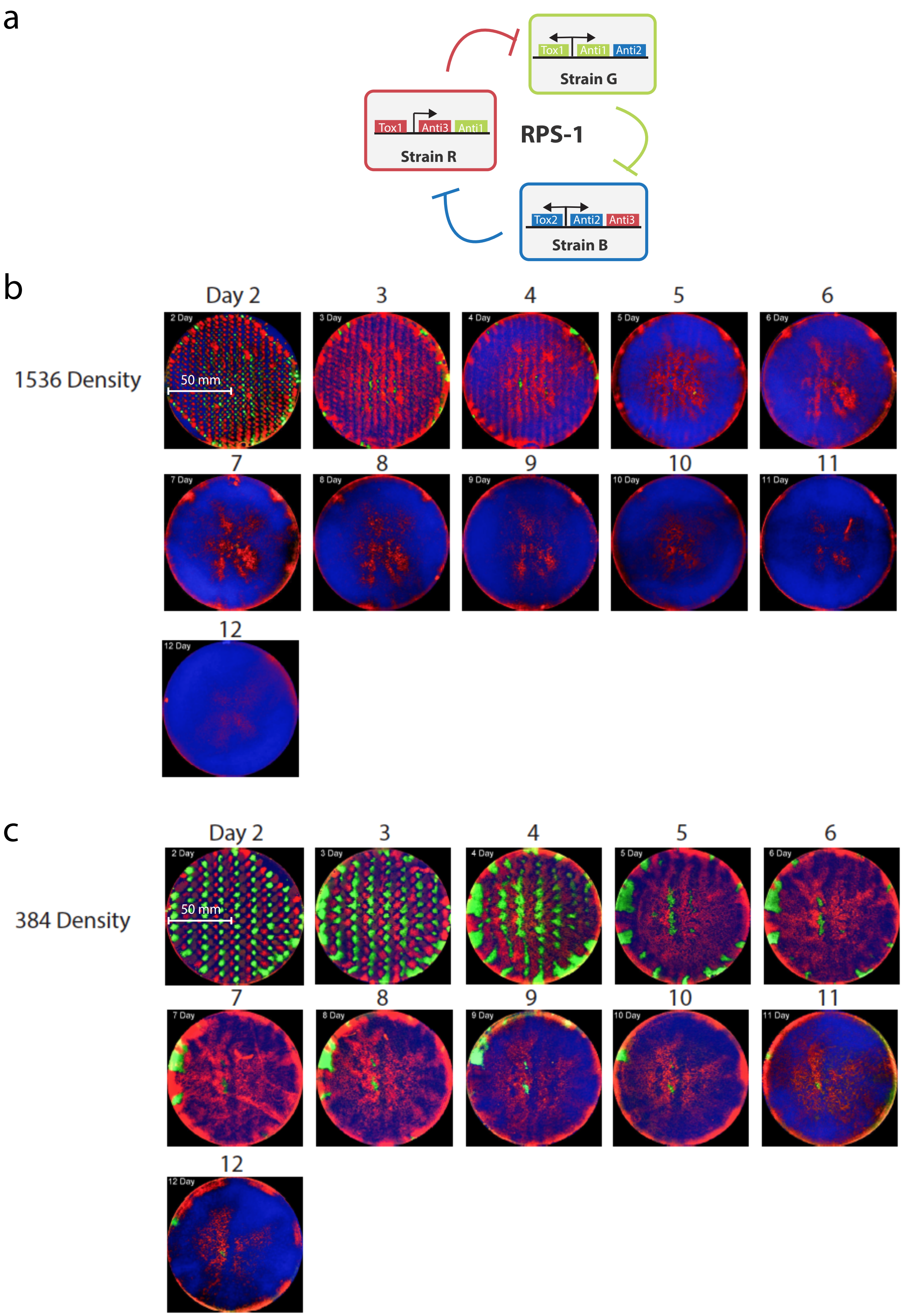

Supplementary Figure 3 | RPS grid passage experiments image stills.(a,) Summary diagram of the competitive interactions among the strains in the RPS-1 community. **(b,)** Image stills of RPS strains arrayed in grid format with a density of 1536 over a period of 12 days. This experiment was executed once at this density. **(c,)** Image stills of RPS strains arrayed in grid format with a density of 384 over a period of 12 days. This experiment was executed once at this density.

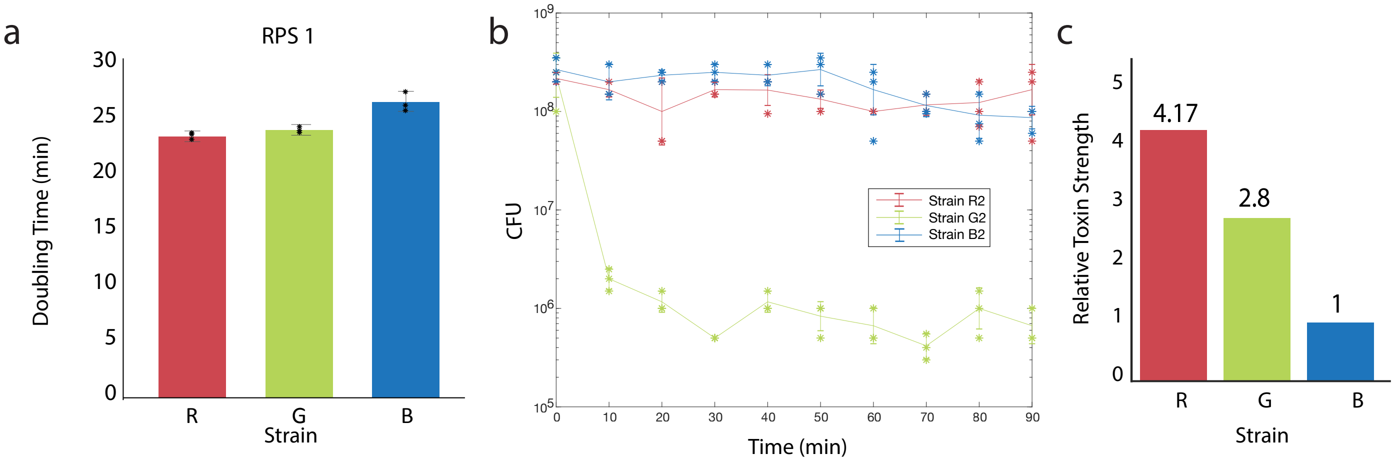

Supplementary Figure 4 | Strain hierarchies for RPS-1. (**a,**) Batch culture growth rates of the engineered RPS 1 E. coli MG1655 strains (n = 3 where n corresponds to the number of separate cell culture replicates inoculated with the each strain and measured independently). All strains were started from the same diluted density and under the same growth conditions. Error bars represent mean +/- standard deviation.(**b,**) Time course results for the liquid kill curve (n = 3 where n is the amount of separate cell cultures replicates for each strain/toxin combination). Error bars are such that the centre value is the geometric mean. The total length of the error bar equals twice the standard deviation error. (**c,**) Toxin strengths of RPS-1 calculated from the kill curves.

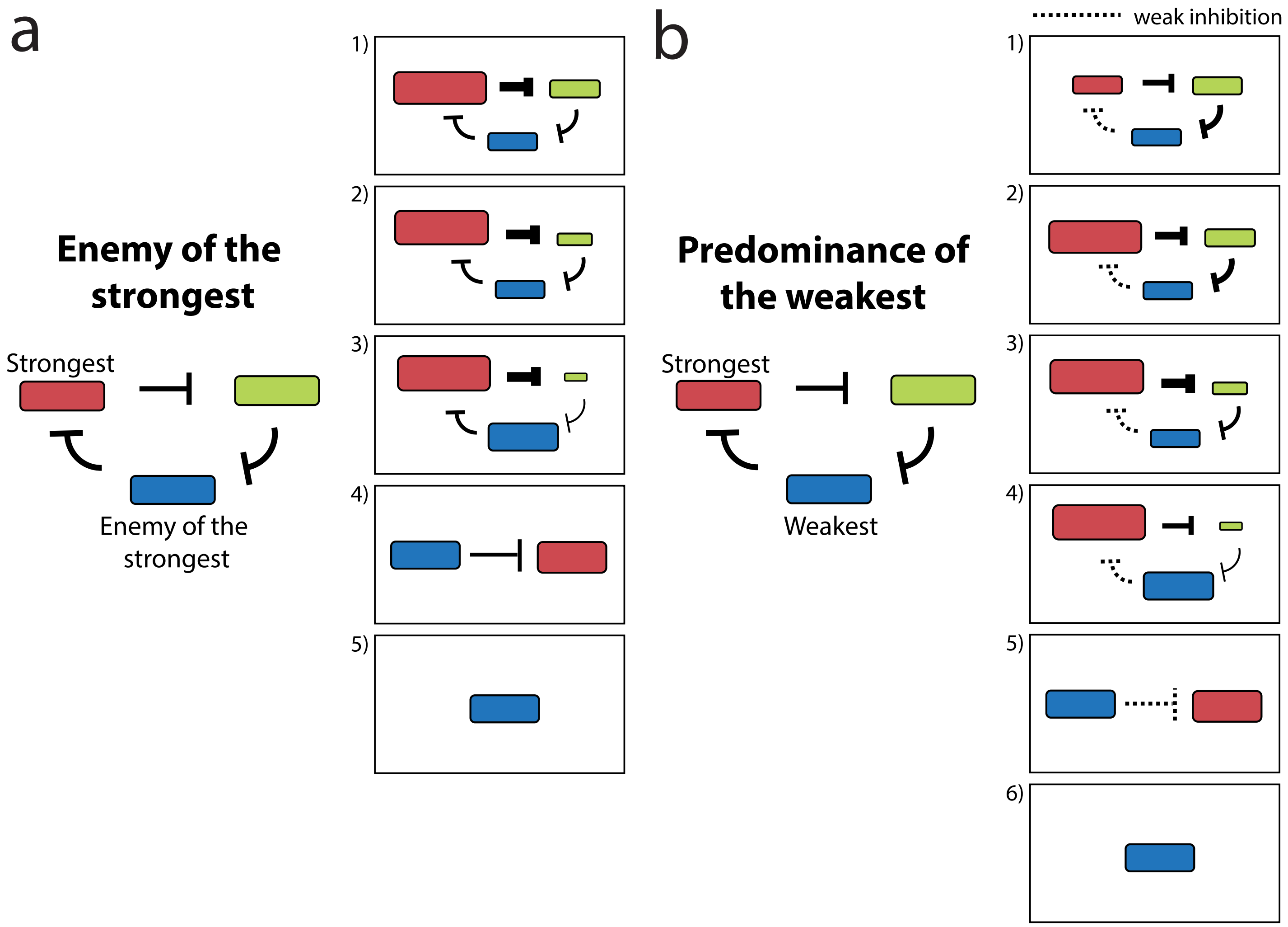

Supplementary Figure 5 | Enemy of the strongest or Predominance of the weakest? (**a,**) Schematic representation of "enemy of the strongest". 1) Strain R is the strongest strain, causing strong inhibition of Strain G. 2) As a result of the strong inhibition, Strain G is eliminated faster relative to the other strains. 3) As Strain G is fully eliminated, Strain B can begin to expand. 4) Strain B slowly outcompetes Strain R. 5) Strain B is the final winner. (**b,**) Schematic representation of "predominance of the weakest". 1) Strain B has very weak inhibition of strain R. 2) As a result, Strain R is able to expand faster relative to the other strains. 3) In response to rapid expansion of Strain R, inhibition of Strain G increases. 4) Eventually, Strain G is fully eliminated, enabling Strain B to grow unchecked. 4) Strain B slowly outcompetes Strain R. 5) Strain B is the final winner.

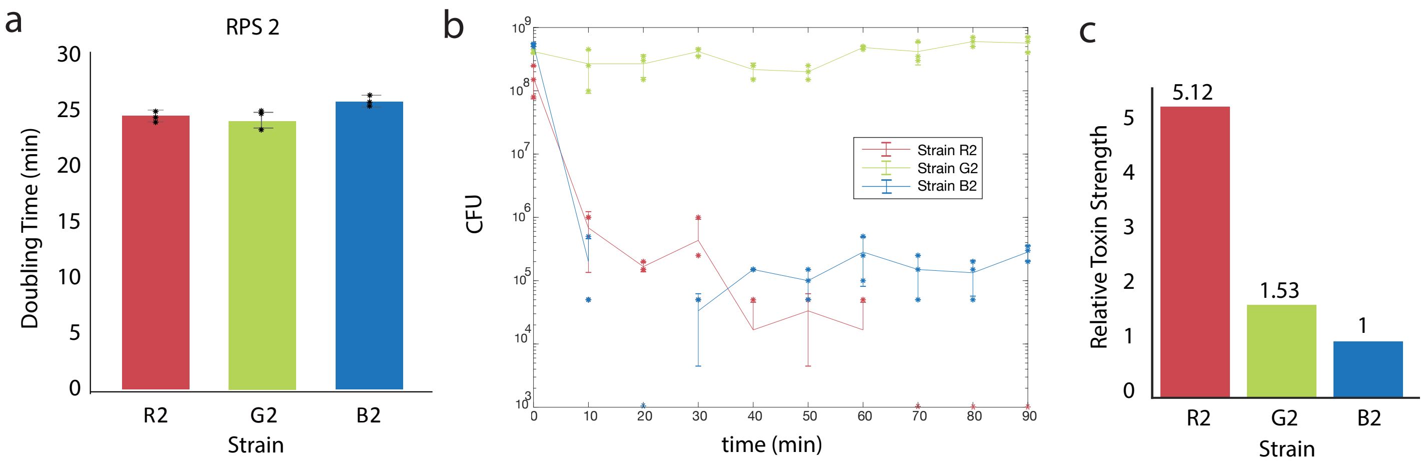

Supplementary Figure 6 | Strain hierarchies for RPS-2. (**a,**) Batch culture growth rates of the engineered RPS 2 E. coli MG1655 strains (n = 3 where n corresponds to the number of separate cell culture replicates inoculated with the each strain and measured independently). All strains were started from the same diluted density and under the same growth conditions. Error bars represent mean +/- standard deviation.(**b,**) Time course results for the liquid kill curve (n = 3 where n is the amount of separate cell cultures replicates for each strain/toxin combination). Error bars are such that the centre value is the geometric mean. The total length of the error bar equals twice the standard deviation error. (**c,**) Toxin strengths of RPS-2 calculated from the kill curves. Time points at which zero colonies were observed are indicated with an asterisk symbol..

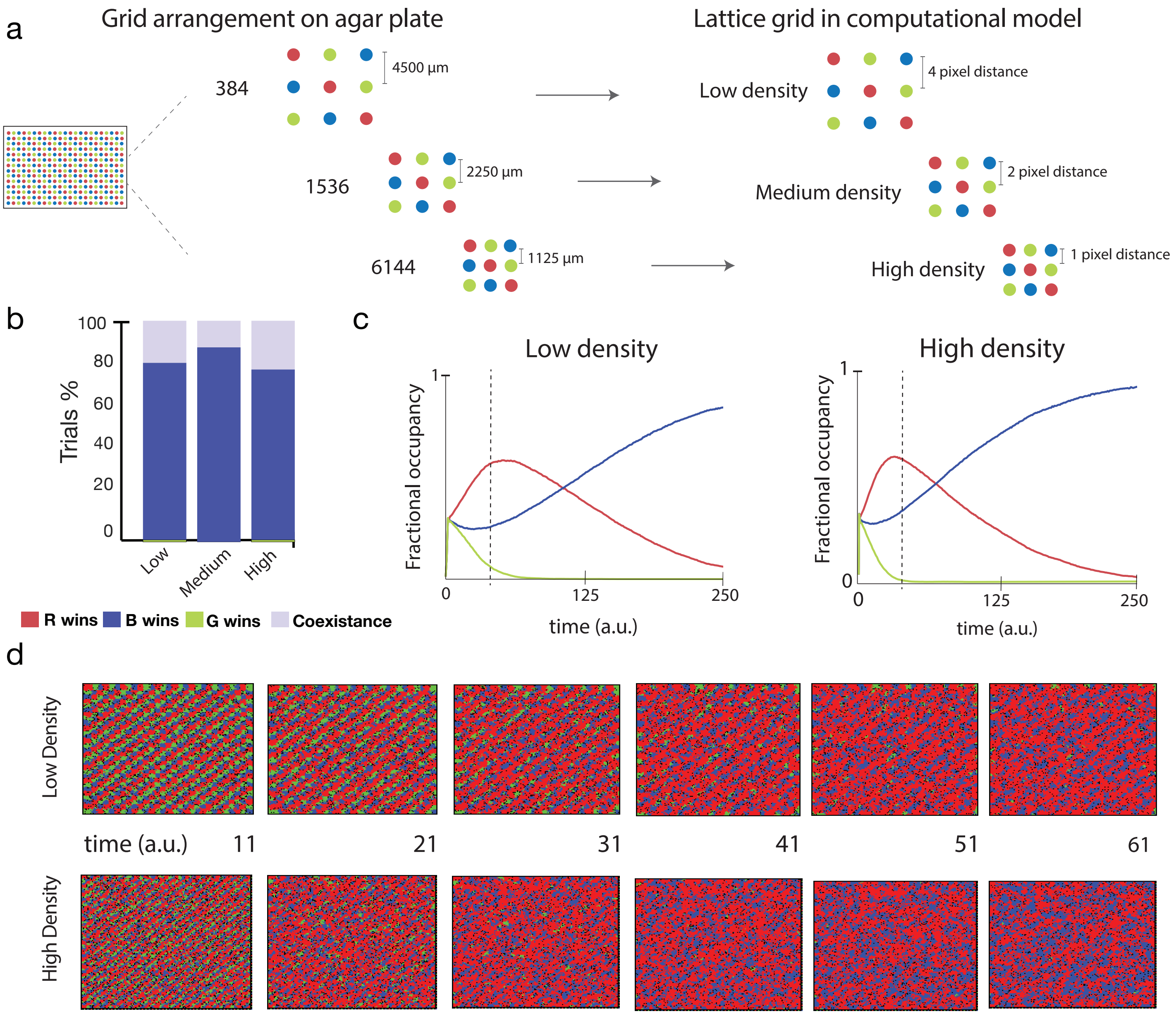

Supplementary Figure 7 | Computational model simulates how initial density affects coexistence. (**a,**) Comparison between the spacing between strains on agar plate (on the left) and in the lattice simulation (on the right). (**b,**) The model was simulated with RPS1 parameters (pR= 0.1, pB= 0.23, pG = 0.42) for different initial densities in grid format. Each condition was simulated 100 times and the steady state value for all runs was summarized in this bar graph. (**c,**) Simulated time series for two simulations starting with different grid densities. (**d,**) Representative frames of the lattice grid at different time points for two different initial densities.

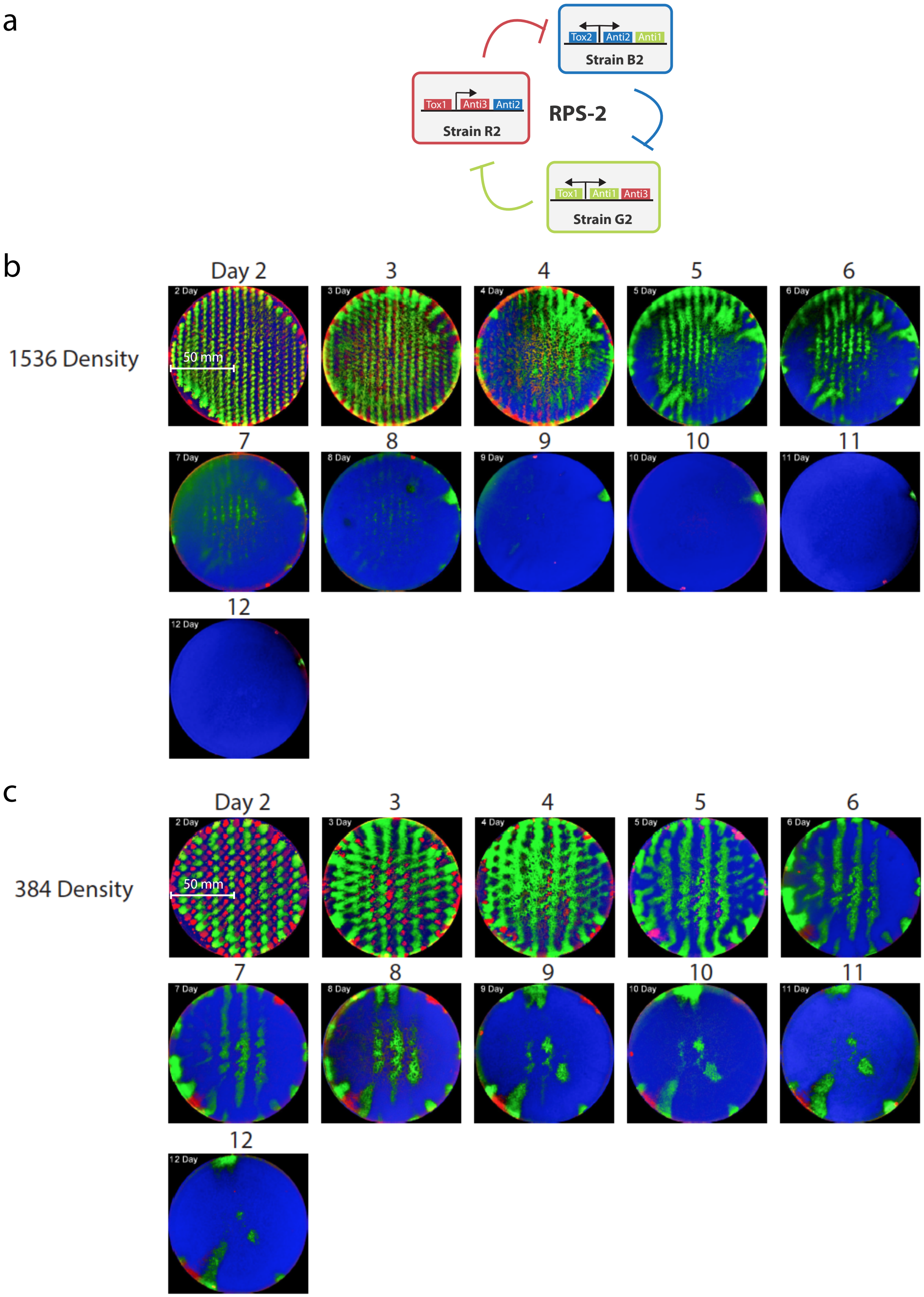

Supplementary Figure 8 | RPS-2 grid passage experiments image stills.(a,) Summary diagram of the competitive interactions among the strains in the RPS-2 community. (**b,**) Image stills of RPS-2 strains arrayed in grid format with a density of 1536 over a period of 12 days. This experiment was executed once at this density.(**c,**) Image stills of RPS-2 strains arrayed in grid format with a density of 384 over a period of 12 days.This experiment was executed once at this density.

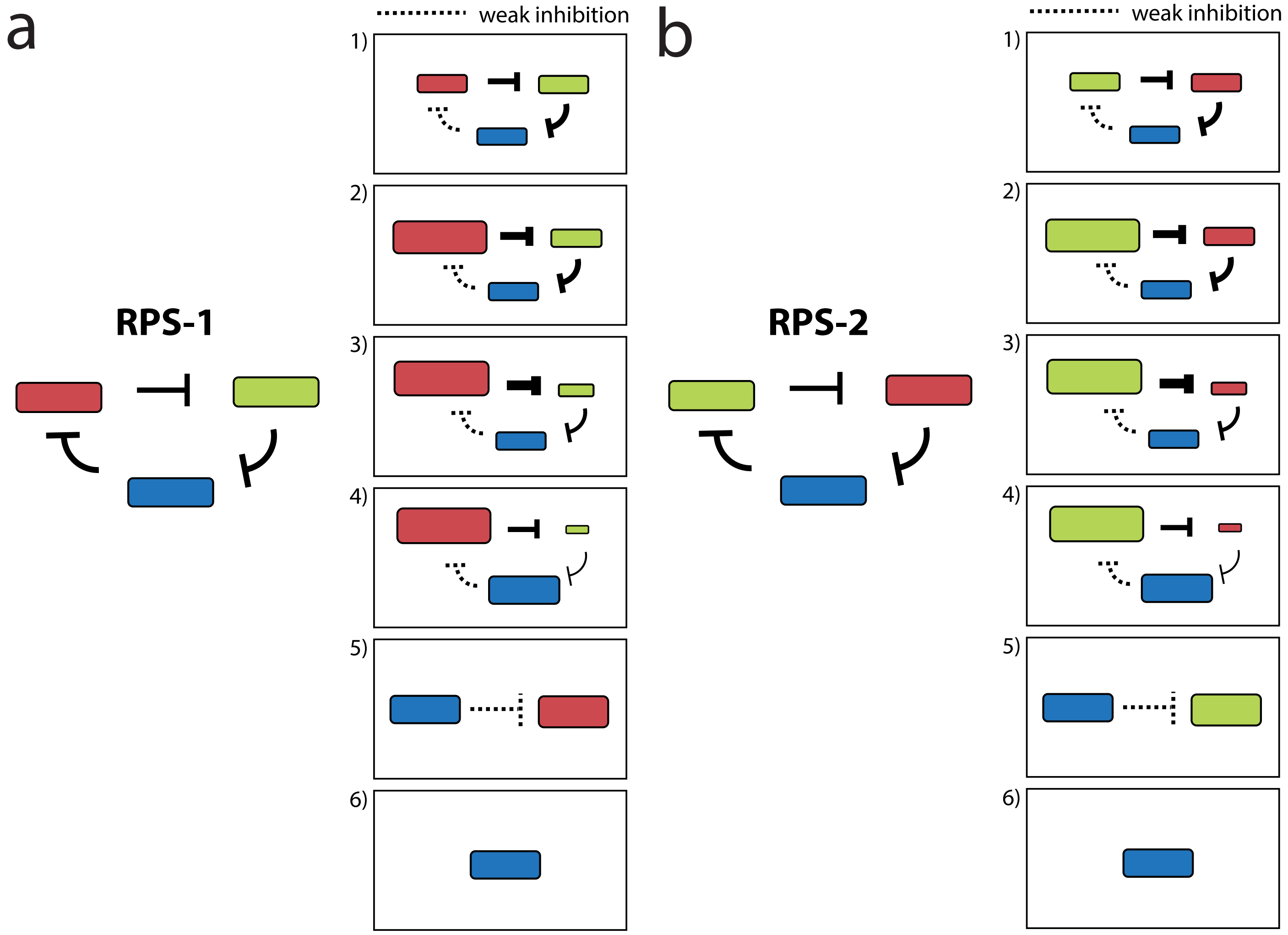

Supplementary Figure 9 | **Predominance of the weakest for RPS-1 and RPS-2** (**a**,) Schematic representation of "predominance of the weakest" for RPS-1. 1) Strain B has very weak inhibition of strain R. 2) As a result, Strain R is able to expand faster relative to the other strains. 3) In response to rapid expansion of Strain R, inhibition of Strain G increases. 4) Eventually, Strain G is fully eliminated, enabling Strain B to grow unchecked. 4) Strain B slowly outcompetes Strain R. 5) Strain B is the final winner. (**b**,) Schematic representation of "predominance of the weakest" for RPS-2. 1) Strain B2 has very weak inhibition of Strain G2. 2) As a result, Strain G2 is able to expand faster relative to the other strains. 3) In response to rapid expansion of Strain G2, inhibition of Strain R2 increases. 4) Eventually, Strain R2 is fully eliminated, enabling Strain B2 to grow unchecked. 4) Strain B2 slowly outcompetes Strain R2. 5) Strain B2 is the final winner.

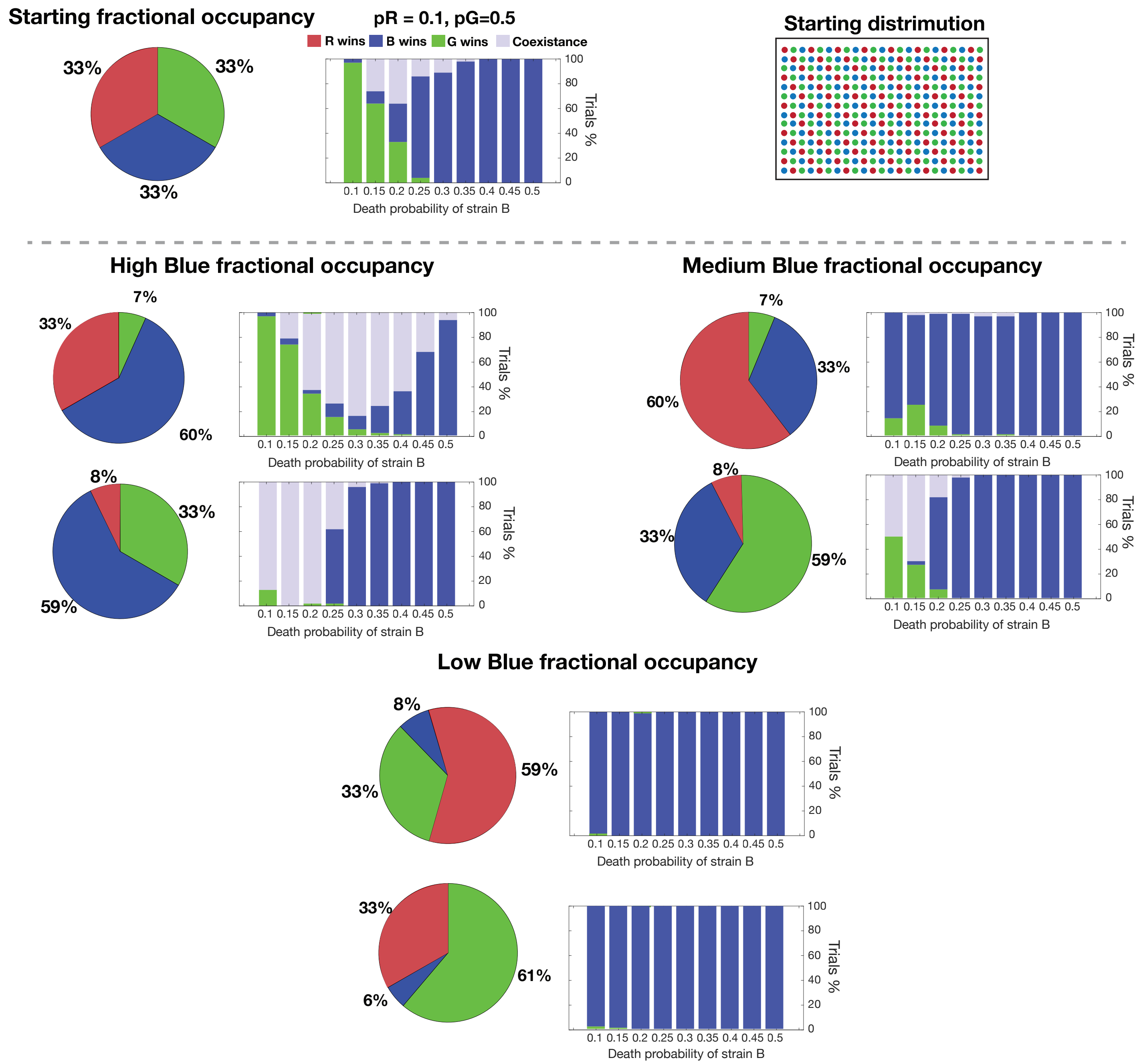

Supplementary Figure 10 | Model simulations explore multiple initial conditions in grid format. Pie charts represent the fractional occupancies of the starting allocation of the three strains. The bar charts show the steady state outcomes of multiple simulations for a range of different toxin strength parameters. The bar plots shows the outcome of 100 trials for multiple parameters of Pb (Probability of death of strain B). For all simulations, the probability of death of strain R (0.1) and strain G (0.5) are kept constant.

Strain Name	Host Bacterium	Plasmid	Referenced in Figure
**Strain R**	**MG1655**	**pML0002** - Colicin E3 + Col E3 Immunity + mKate2 + Col E7 Immunity + Col E1 Lysis Protein	*1a, 1b, 2a-f, 3a-c, 4a-e, Extended Data Figs 1-4*
**Strain G**	**MG1655**	**pML0003** - Colicin E7 + Col E7 Immunity + sfGFP + Col V Immunity + Col E1 Lysis Protein	*1a, 1b, 2a-f, 3a-c, 4a-e, Extended Data Figs 1-4*
**Strain B**	**MG1655**	**pML0137** - Colicin V + Colicin V Immunity + Colicin V secretion protein (*CvaA*) + Colicin V secretion/processing ATP-bindingn protein (*CvaB*) + Col E3 Immunity	*1a, 1b, 2a-f, 3a-c, 4a-e, Extended Data Figs 1-4*
**Strain R2**	**MG1655**	**pML0004** - Colicin E3 + Col E3 Immunity + mKate2 + Col V Immunity + Col E1 Lysis Protein	*3a-c, Extended Data Figs 6,8*
**Strain G2**	**MG1655**	**pML0001** - Colicin E7 + Col E7 Immunity + sfGFP + Col E3 Immunity + Col E1 Lysis Protein	*3a-c, Extended Data Figs 6,8*
**Strain B2**	**MG1655**	**pML0146** - Colicin V + Colicin V Immunity + Colicin V secretion protein (*CvaA*) + Colicin V secretion/processing ATP-bindingn protein (*CvaB*) + Col E7 Immunity	*3a-c, Extended Data Figs 6,8*

Supplementary Table 1 | The strains used in this study. This table summarizes the gene content of each plasmid mentioned in this study, as well as the host chassis used and the respective figures in which they are referenced.

Protein/Gene	AA Sequence
**E1 Lysis**	MRKRFFVGIFAINLLVGCQANYIPDVQGGTIAPSSSSKLTGIAVQ*
**Colicin E3**	MSGGDGRGHNTGAHSTSGNINGGPTGLGVGGGASDGSGWSSENNPWGGGSGSGIHWGGGSGHGNGGGNGNSGGGSGTGGNL<br>SAVAAPVAFGFPALSTPGAGGLAVSISAGALSAAIADIMAALKGPFKFGLWGVALYGVLPSQIAKDDPNMMSKIVTSLPADDITESPVSS<br>LPLDKATVNVNVRVVDDVKDERQNISVVSGVPMSVPVVDAKPTERPGVFTASIPGAPVLNISVNNSTPAVQTLSPGVTNNTDKDVRPA<br>GFTQGGNTRDAVIRFPKDSGHNAVYVSVSDVLSPDQVKQRQDEENRRQQEWDATHPVEAAERNYERARAELNQANEDVARNQER<br>QAKAVQVYNSRKSELDAANKTLADAIAEIKQFNRFAHDPMAGGHRMWQMAGLKAQRAQTDVNNKQAAFDAAAKEKSDADAAL<br>SSAMESRKKKEDKKRSAENNLNDEKNKPRKGFKDYGHDYHPAPKTENIKGLGDLKPGIPKTPKQNGGGKRKRWTGDKGRKIYEWDS<br>QHGELEGYRASDGQHLGSFDPKTGNQLKGPDPKRNIKKYL*
**Col E3 Immunity**	MGLKLDLTWFDKSTEDFKGEEYSKDFGDDGSVMESLGVPFKDNVNNGCFDVIAEWVPLLQPYFNHQIDISDNEYFV<br>SFDYRDGDW*
**Colicin E7**	MSGGDGRGHNSGAHNTGGNINGGPTGLGGNGGASDGSGWSSENNPWGGGSGSGVHWGGGSGHGNGGGNSN<br>SGGGSNSSVAAPMAFGFPALAAPGAGTLGISVSGEALSAAIADIFAALKGPFKFSAWGIALYGILPSEIAKDDPNMMSK<br>IVTSLPAETVTNVQVSTLPLDQATVSVTKRVTDVVKDTRQHIAVVAGVPMSVPVVNAKPTRTPGVFHASFPGVPSLTVS<br>TVKGLPVSTTLPRGITEDKGRTAVPAGFTFGGGSHEAVIRFPKESGQKPVYVSVTDVLTPAQVKQRQDEEKRLQQEWN<br>DAHPVEVAERNYEQARAELNQANKDVARNQERQAKAVQVYNSRKSELDAANKTLADAKAEIKQFERFAREPMAAGH<br>RMWQMAGLKAQRAQTDVNNKKAAFDAAAKEKSDADVALSSALERRKQKENKEKDAKAKLDKESKRNKPGKATGKG<br>KPVNNKWLNNAGKDLGSPVPDRIANKLRDKEFKSFDDFRKKFWEEVSKDPELSKQFSRNNNDRMKVGKAPKTRTQD<br>VSGKRTSFELHHEKPISQNGGVYDMDNISVVTPKRHIDIHRGK*
**Col E7 Immunity**	MELKNSISDYTEAEFVQLLKEIEKENVAATDDVLDVLLEHFVKITEHPDGTDLIYYPSDNRDDSPEGIVKEIKEWRAANG<br>KPGFKQG*
**CvaC**	MRTLTLNELDSVSGGASGRDIAMAIGTLSGQFVAGGIGAAAGGVAGGAIYDYASTHKPNPAMSPSGLGGTIKQKPEGI<br>PSEAWNYAAGRLCNWSPNNLSDVCL*
**Cvi**	MDRKRTKLELLFAFIINATAIYIALAIYDCVFRGKDFLSMHTFCFSALMSAICYFVGDNYYSISDKIKRRSYENSDSK*
**CvaA**	MKWQGRAILLPGIPLWLIMLGSIVFITAFLMFIIVGTYSRRVNVSGEVTTWPRAVNIYSGVQGFVVRQFVHEGQLIKKGD<br>PVYLIDISKSTRNGIVTDNHRRDIENQLVRVDNIISRLEESKKITLDTLEKQRLQYTDAFRRSSDIIQRAEEGIKIMKNNMEN<br>YRYYQSKGLINKDQLTNQVALYYQQQNNLLSLSGQNEQNALQITTLESQIQTQAADFDNRIYQMELQRLELQKELVNT<br>DVEGEIIIRALSDGKVDSLSVTVGQMVNTGDSLLQVIPENIENYYLILWVPNDAVPYISAGDKVNIRYEAFPSEKFGQFSA<br>TVKTISRTPASTQEMLTYKGAPQNTPGASVPWYKVIATPEKQIIRYDEKYLPLENGMKAESTLFLEKRRIYQWMLSPFYD<br>MKHSATGPIND*
**CvaB**	MTNRNFRQIINLLDLRWQRRVPVIHQTETAECGLACLAMICGHFGKNIDLIYLRRKFNLSARGATLAGINGIAEQLGMA<br>TRALSLELDELRVLKTPCILHWDFSHFVVLVSVKRNRYVLHDPARGIRYISREEMSRYFTGVALEVWPGSEFQSETLQTRI<br>SLRSLINSIYGIKRTLAKIFCLSVVIEAINLLMPVGTQLVMDHAIPAGDRGLLTLISAALMFFILLKAATSTLRAWSSLVMSTL<br>INVQWQSGLFDHLLRLPLAFFERRKLGDIQSRFDSLDTLRATFTTSVIGFIMDSIMVVGVCVMMLLYGGYLTWIVLCFTT<br>IYIFIRLVTYGNYRQISEECLVREARAASYFMETLYGIATVKIQGMVGIRGAHWLNMKIDAINSGIKLTRMDLLFGGINTFV<br>TACDQIVILWLGAGLVIDNQMTIGMFVAFSSFRGQFSERVASLTSFLLQLRIMSLHNERIADIALHEKEEKKPEIEIVADMG<br>PISLETNGLSYRYDSQSAPIFSALSLSVAPGESVAITGASGAGKTTLMKVLCGLFEPDSGRVLINGIDIRQIGINNYHRMIA<br>CVMQDDRLFSGSIRENICGFAEEMDEEWMVECARASHIHDVIMNMPMGYETLIGELGEGLSGGQKQRIFIARALYRKP<br>GILFMDEATSALDSESEHFVNVAIKNMNITRVIIAHRETTLRTVDRVISI*

Supplementary Table 2 | Genes protein sequences. This table includes the amino acid sequences of all the toxins, immunity and lysis proteins used in this study. The symbol * represents a STOP codon.

Parameter Name	Value	Correspondence
Lattice size	150 x 150 pixels	Simulates the surface of a petri dish of size 15 cm
Distance between colonies (low density)	4 pixels	Corresponds to 4500μm arrayed with the Echo
Distance between colonies (medium density)	2 pixels	Corresponds to 2250μm arrayed with the Echo
Distance between colonies (high density)	1 pixel	Corresponds to 1125μm arrayed with the Echo
Minimum probability of death	0.05	Represents the stochastic death/removal of cells
Maximum probability of death strain R	0.1	Calculated according to the kill curve results
Maximum probability of death strain B	0.28	Calculated according to the kill curve results
Maximum probability of death strain G	0. 417	Calculated according to the kill curve results
Maximum probability of death strain R2	0.153	Calculated according to the kill curve results
Maximum probability of death strain B2	0.512	Calculated according to the kill curve results
Maximum probability of death strain R2	0.1	Calculated according to the kill curve results

Supplementary Table 3 | Model parameters list. This table includes the list of parameters used in the computational model with their respective values and significance.
