# Supplementary material for: Survival of the weakest in non-transitive asymmetric interactions among strains of E. coli
Source: Nat Commun. 2020 Nov 27;11:6055. doi: 10.1038/s41467-020-19963-8 (PMC7699631; doi:10.1038/s41467-020-19963-8)
Supplement: Supplementary file 3 — Description of Additional Supplementary Files [file 41467_2020_19963_MOESM3_ESM.pdf]

Supplementary Movie 1
Image sequence of fluorescent agar plate images for RPS-1 at a 1:1000 dilution. Strains were initially seeded at a 1:1:1 ratio of Strain R, G, B randomly mixed. Images were taken every 24 hours.

Supplementary Movie 2
Image sequence of fluorescent agar plate images for RPS-1 at a 1:10000 dilution. Strains were initially seeded at a 1:1:1 ratio of Strain R, G, B randomly mixed. Images were taken every 24 hours.

Supplementary Movie 3
Lattice model simulation of RPS-1 in a medium density grid. Toxin strength parameters derived from liquid kill curve experimental results. Probability of death of Strain R, B, G was 0.1, 0.28, and 0.417 respectively.

Supplementary Movie 4
Lattice model simulation of RPS-2 in a medium density grid. Toxin strength parameters derived from liquid kill curve experimental results. Probability of death of Strain R, B, G was 0.153, 0.512, and 0.1 respectively.

Supplementary Movie 5
Image sequence of fluorescent agar plate images for RPS-1 (left) and RPS-2 (right) initially spotted in a grid format. The top row shows low density initial seeding. The bottom row shows medium density initial seeding. Images were taken every 24 hours.

Supplementary Movie 6
Lattice model simulation of RPS-1 in a medium density grid. Toxin strength parameters derived from liquid kill curve experimental results. Probability of death of Strain R, B, G was 0.1, 0.2, and 0.5 respectively.

Supplementary Movie 7
Lattice model simulation demonstrating one of the possible scenarios with RPS-1 initially distributed in vertical stripes. Toxin strength parameters derived from liquid kill curve experimental results. Probability of death of Strain R, B, G was 0.1, 0.28, and 0.417 respectively.

Supplementary Movie 8
Lattice model simulation demonstrating one of the possible scenarios with RPS-1 initially distributed in separate blocks. Toxin strength parameters derived from liquid kill curve experimental results. Probability of death of Strain R, B, G was 0.1, 0.28, and 0.417 respectively.

Supplementary Movie 9

Lattice model simulation demonstrating one of the possible scenarios with RPS-1 initially distributed in concentric circles with the strongest strain in the middle. In this example, the central circle has a smaller radius compared to the thickness of the other rings. Toxin strength parameters derived from liquid kill curve experimental results. Probability of death of Strain R, B, G was 0.1, 0.28, and 0.417 respectively.

Supplementary Movie 10

Lattice model simulation demonstrating one of the possible scenarios with RPS-1 initially distributed in concentric circles with the strongest strain in the middle. Toxin strength parameters derived from liquid kill curve experimental results. Probability of death of Strain R, B, G was 0.1, 0.28, and 0.417 respectively.
