# Supplementary material for: Survival of the weakest in non-transitive asymmetric interactions among strains of E. coli
Source: Nat Commun. 2020 Nov 27;11:6055. doi: 10.1038/s41467-020-19963-8 (PMC7699631; doi:10.1038/s41467-020-19963-8)
Supplement: Supplementary file 14 — Reporting Summary [file 41467_2020_19963_MOESM14_ESM.pdf]

nature research

Corresponding author(s): Jeff Hasty

Last updated by author(s): Oct 12, 2020

Reporting Summary

Nature Research wishes to improve the reproducibility of the work that we publish. This form provides structure for consistency and transparency in reporting. For further information on Nature Research policies, see our Editorial Policies and the Editorial Policy Checklist.

Statistics

For all statistical analyses, confirm that the following items are present in the figure legend, table legend, main text, or Methods section.

n/a	Confirmed	
☐	☒	The exact sample size (n) for each experimental group/condition, given as a discrete number and unit of measurement
☐	☒	A statement on whether measurements were taken from distinct samples or whether the same sample was measured repeatedly
☒	☐	The statistical test(s) used AND whether they are one- or two-sided<br>*Only common tests should be described solely by name; describe more complex techniques in the Methods section.*
☒	☐	A description of all covariates tested
☒	☐	A description of any assumptions or corrections, such as tests of normality and adjustment for multiple comparisons
☐	☒	A full description of the statistical parameters including central tendency (e.g. means) or other basic estimates (e.g. regression coefficient) AND variation (e.g. standard deviation) or associated estimates of uncertainty (e.g. confidence intervals)
☒	☐	For null hypothesis testing, the test statistic (e.g. F, t, r) with confidence intervals, effect sizes, degrees of freedom and P value noted<br>*Give P values as exact values whenever suitable.*
☒	☐	For Bayesian analysis, information on the choice of priors and Markov chain Monte Carlo settings
☒	☐	For hierarchical and complex designs, identification of the appropriate level for tests and full reporting of outcomes
☒	☐	Estimates of effect sizes (e.g. Cohen's d, Pearson's r), indicating how they were calculated

Our web collection on statistics for biologists contains articles on many of the points above.

Software and code

Policy information about availability of computer code

Data collection	ImageJ 2.0.0-rc-69/1.52p was used for data extraction from microscopy images. Simulation data was obtained through custom code written in Matlab R2019a.
Data analysis	Images were processed using ImageJ 2.0.0-rc-69/1.52p.

For manuscripts utilizing custom algorithms or software that are central to the research but not yet described in published literature, software must be made available to editors and reviewers. We strongly encourage code deposition in a community repository (e.g. GitHub). See the Nature Research guidelines for submitting code & software for further information.

Data

Policy information about availability of data

All manuscripts must include a data availability statement. This statement should provide the following information, where applicable:

- Accession codes, unique identifiers, or web links for publicly available datasets
- A list of figures that have associated raw data
- A description of any restrictions on data availability

Authors can confirm that all relevant data are included in the paper and/or its supplementary information files. All other data, plasmids and bacterial strains are available from the corresponding author upon request.

Field-specific reporting

Please select the one below that is the best fit for your research. If you are not sure, read the appropriate sections before making your selection.

- [x] Life sciences
- [ ] Behavioural & social sciences
- [ ] Ecological, evolutionary & environmental sciences

For a reference copy of the document with all sections, see nature.com/documents/nr-reporting-summary-flat.pdf

Life sciences study design

All studies must disclose on these points even when the disclosure is negative.

Sample size	For the randomly distributed agar plate experiments we used a sample size of n=5. These samples consisted of serial diluted strain compositions covering a range from no dilution (resulting in a fully grown lawn of bacteria after 24 hours) to a 10^-4 dilution (resulting in only a few starting colonies). From this experiment we observed that the outcome of the competition remained the same regardless of initial starting density. For the ordered grid agar plate experiments we used a sample size of n=2 due to limitations in our liquid handling robot. Using the liquid handling robot the samples were plated at 2 different grid densities corresponding to 384, and 1536 well plate spacing. The orders and concentrations among the three strains were kept consistent in each density. Due to the consistent outcomes among the randomly distributed plates (n=5) as well as the ordered grid plates (n=2) we believe the sample sizes were sufficient.
Data exclusions	No data was excluded.
Replication	For the randomly distributed agar plate experiments, the serially diluted strain compositions were plated on 5 different agar plates corresponding a range from no dilution to a 10^-4 dilution in order to create 5 replicates for each ratio of strains. For the ordered grid agar plate experiments, the samples were plated at 3 different grid densities corresponding to 384, and 1536 well plate spacing. The orders and concentrations among the three strains were kept consistent in each density in order to create 2 replicates.
Randomization	Due to the initial conditions such as starting density and strain composition, and visual nature of imaging bacterial colonies based on fluorescence expression, randomization was not used.
Blinding	The outcome of bacterial competition was monitored and imaged using fluorescent biomarkers. Due to the nature of this study, blinding was not considered.

Reporting for specific materials, systems and methods

We require information from authors about some types of materials, experimental systems and methods used in many studies. Here, indicate whether each material, system or method listed is relevant to your study. If you are not sure if a list item applies to your research, read the appropriate section before selecting a response.

Materials & experimental systems

n/a	Involved in the study
x	Antibodies
x	Eukaryotic cell lines
x	Palaeontology and archaeology
x	Animals and other organisms
x	Human research participants
x	Clinical data
x	Dual use research of concern

Methods

n/a	Involved in the study
x	ChIP-seq
x	Flow cytometry
x	MRI-based neuroimaging
